# Intracellular albumin overload elicits endoplasmic reticulum stress and PKC-delta/p38 MAPK pathway activation to induce podocyte apoptosis

**DOI:** 10.1038/s41598-018-36933-9

**Published:** 2018-12-20

**Authors:** Guilherme Lopes Gonçalves, Juliana Martins Costa-Pessoa, Karina Thieme, Bruna Bezerra Lins, Maria Oliveira-Souza

**Affiliations:** Laboratory of Renal Physiology, Department of Physiology and Biophysics, Institute of Biomedical Sciences, University of Sao Paulo, Sao Paulo, Brazil

## Abstract

Podocyte injury is closely related to proteinuria and the progression of chronic kidney disease (CKD). Currently, there is no conclusive understanding about the mechanisms involved in albumin overload and podocyte apoptosis response. In this study, we sought to explore the ways by which intracellular albumin can mediate podocyte apoptosis. Here, immortalized mouse podocytes were treated with bovine serum albumin (BSA) at different times and concentrations, in the presence or absence of SB203580 (0.1 µM, inhibitor of mitogen-activated-protein kinase – p38MAPK). Using immunofluorescence images, flow cytometry and immunoblotting, we observed a time-dependent intracellular accumulation of fluorescent albumin-FITC-BSA, followed by concentration-and time-dependent effect of intracellular albumin overload on podocyte apoptosis, which was mediated by increased expression of the chaperone glucose-regulated-protein 78 (GRP 78) and phosphorylated inositol-requiring enzyme 1 alpha (pIRE1-α), as well as protein kinase C delta (PKC-δ), p38MAPK and cleaved caspase 12 expression. SB203580 prevented the cleavage of caspase 12 and the albumin-mediated podocyte apoptosis. These results suggest that intracellular albumin overload is associated with endoplasmic reticulum (ER) stress and upregulation of PKC-δ/p38MAPK/caspase 12 pathway, which may be a target for future therapeutic of albumin-induced podocyte apoptosis.

## Introduction

Podocytes are highly specialized renal epithelial cells in the Bowman’s space. They regularly wrap around the glomerular basement membrane (GBM) of the glomerular capillaries and extend foot processes, which interdigitate with the same structures of the neighboring podocytes. The extracellular domains of specific integral membrane proteins, including nephrin, p-cadherin and FAT tumor suppressor homolog 1 (FAT 1) extend between the foot processes to form the slit diaphragm^1,2^, which under physiological conditions, forms a selective barrier that is permeable to water and small solutes but has limited permeability to macromolecules such as albumin^3^.

On the other hand, in renal diseases, podocytes are injured and the complex architecture of the foot processes can be altered, leading to loss of the slit diaphragm or podocyte effacement. These processes result in proteinuria and progressive loss of kidney function^4,5^. During podocyte dysfunction, large amounts of albumin are found in the glomerular ultrafiltrate; however they are predominantly reabsorbed by endocytosis in proximal tubular cells^6^. In addition to this classical pathway, it has been demonstrated that podocytes can endocyte proteins such as albumin^7^. Furthermore, studies in human and animal models have reported a transcellular migration of albumin through the podocytes under albuminuric conditions^6–10^, and albumin overload in podocytes results in increased expression of the GRP 78, which has been reported as an ER stress marker^11,12^. However, the mechanisms by which intracellular albumin induces ER-stress-mediated podocyte injury are not well understood.

It has been established that in vascular smooth muscle cells, adipocytes or the Human Embryonic Kidney 293 (HEK293) cells, nicotinamide adenine dinucleotide phosphate (NADPH) oxidase stimulation and reactive oxygen species (ROS) production are closely related to endoplasmic reticulum (ER) stress, PKC-δ^13–15^ and p38 MAPK activation, which together are associated with apoptotic responses^13,16–18^.

In view of these findings, we sought to explore the hypothesis that under intracellular albumin overload, the ER stress/PKC-δ/p38MAPK/caspase 12 signaling pathway can play a critical role in podocyte apoptosis. Our data advance the understanding of the cell signaling pathways responsible for albumin-induced podocyte apoptosis and may contribute to the development of preventive and therapeutic strategies for albuminuria-associated chronic kidney diseases (CKD).

## Results

### Expression of podocin and synaptopodin

**Figure 1:**
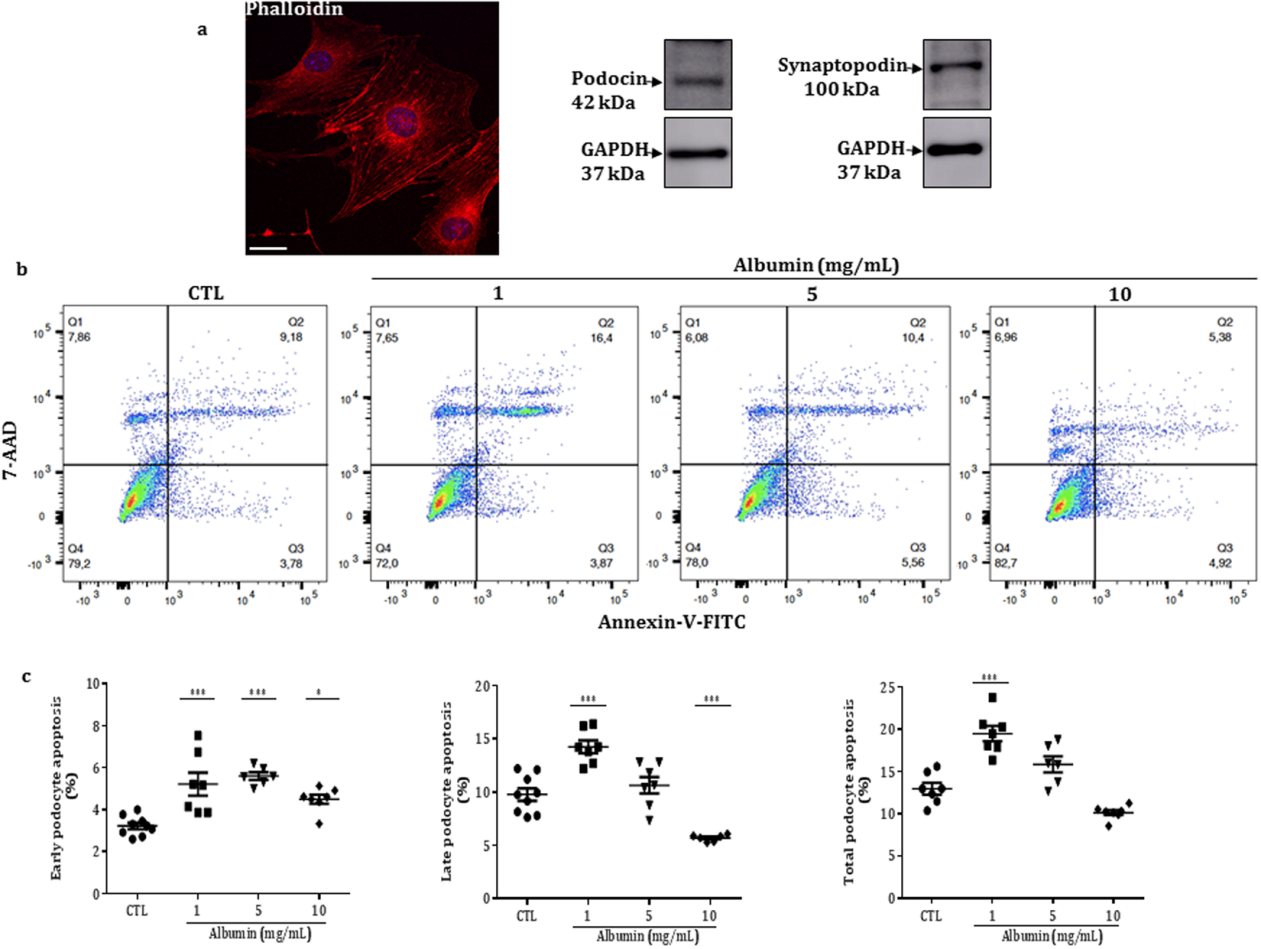
(**a**) Representative immunofluorescence and immunoblots from 4 experiments with differentiated podocytes under control conditions. Phalloidin images were captured on a Zeiss LSM 780 confocal microscope equipped with a 63× objective plan-apochromat zoom factor 1 and a laser excitation of 546 nm for phalloidin. Bar, 20 μm. Immunoblot analysis results are presented for the podocin and synaptopodin protein expression, as well as the internal control glyceraldehyde-3-phosphate dehydrogenase (GAPDH). (**b**) Representative flow cytometry data exhibiting podocyte apoptosis in the control or treated groups. The cells were treated with BSA (1, 5 and 10 mg/mL) for 24 hours, and apoptosis was evaluated by flow cytometry using FITC Annexin V/7-AAD. Q1, cells in necrosis; Q2, cells in late apoptosis; Q3, cells in early apoptosis and Q4, healthy cells. (**c**) The values in percentage (%) are expressed as mean ± SEM of 6–9 experiments in triplicate.

To validate our *in vitro* model, differentiated mouse podocytes were subjected to phalloidin immunofluorescence staining and podocin and synaptopodin protein expression (Fig. 1a).

### Albumin overload induces podocyte apoptosis

Differentiated podocytes were cultured in BSA-free medium or with increasing concentrations of BSA (1, 5 or 10 mg/mL) at 37 °C for 24 hours. Under these conditions, podocyte apoptosis was documented by flow cytometry. The treatment with albumin 1 mg/mL induced significant increase of the early, late and consequently total podocyte apoptosis compared to control (untreated) cells. Albumin 5 mg/mL induced only early podocyte apoptosis, whereas albumin 10 mg/mL induced a significant increase in the early apoptosis but decreased the late apoptosis. Thus, in these conditions, total podocyte apoptosis did not change compared to the control group (Fig. 1b,c and Table 1).

**Table 1:** Effect of albumin on apoptosis in control and treated podocytes.

Apoptosis, %	Control	Alb −1 mg/mL	Alb −5 mg/mL	Alb −10 mg/mL
Early	3. 23 ± 0.15 (9)	5.22 ± 0.54^***^ (7)	5.60 ± 0.17^***^(6)	4.50 ± 0.21^*^ (7)
Late	9. 78 ± 0.58 (9)	14.25 ± 0.60 ^***^(7)	10.65 ± 0.77 (7)	5.70 ± 0.12 ^***^(6)
Total	12.95 ± 0.69 (7)	19.47 ± 0.90^***^ (7)	15.83 ± 0.96 (6)	10.12 ± 0.30 (7)

The values are mean ± SEM; Number of experiments in parentheses. ^***^p < 0.001 and ^*^p < 0.05 *versus* control (untreated), Alb, albumin.

### Time and temperature-dependence of the FITC-BSA internalization in podocyte

**Figure 2:**
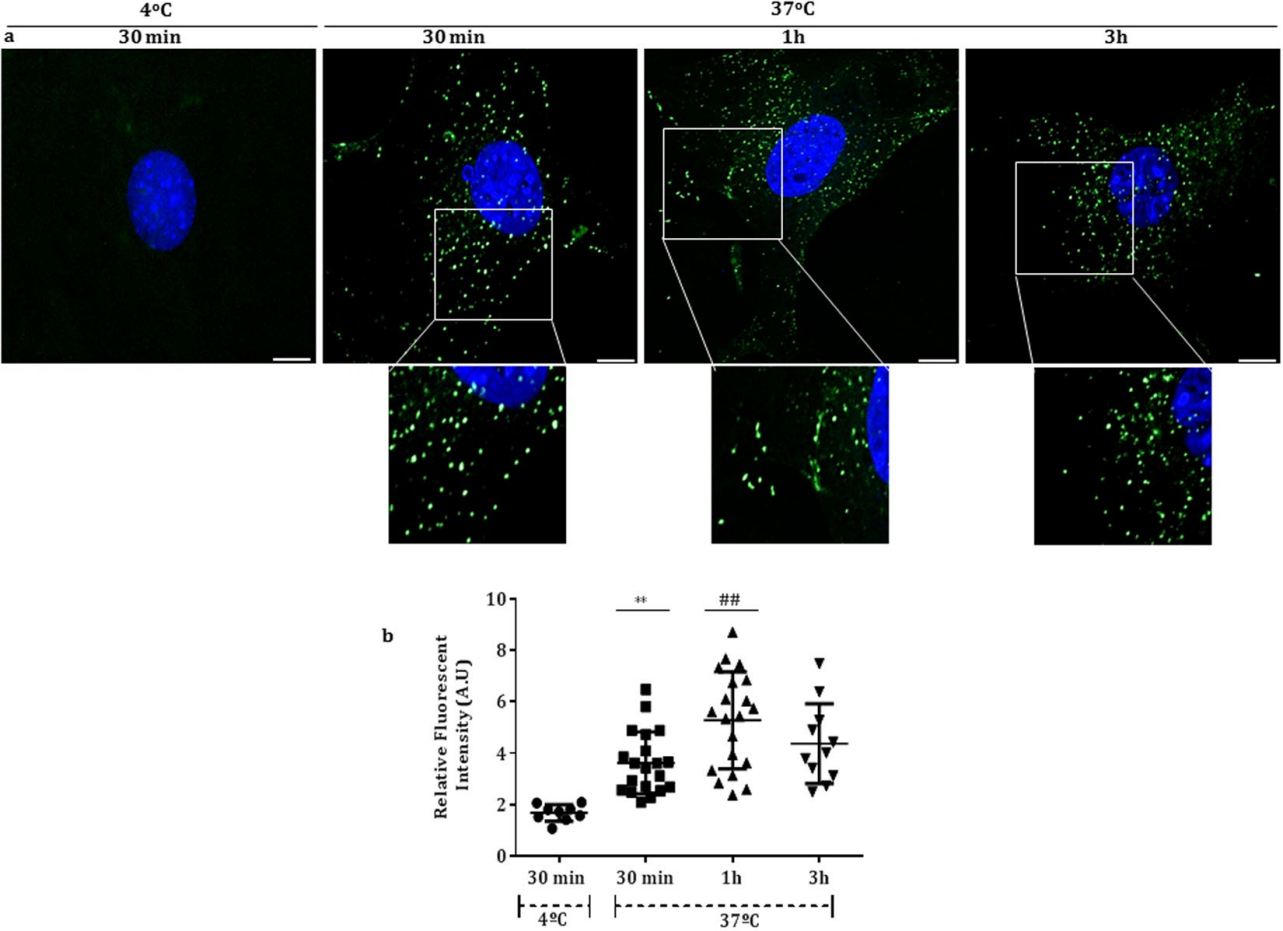
(**a**) Fluorescent images demonstrating that the albumin internalization in podocytes is temperature and time-dependent. The signal was detected as green vesicles distributed in the cytosol and enlarged images are also represented. Bar, 20 μm. (**b**) The fluorescence intensity was expressed as arbitrary units (A.U) of 9–20 cells.

We monitored the albumin internalization after stimulation with FITC-BSA (1 mg/mL in serum free medium) at 4 °C for 30 minutes and at 37 °C for 30 minutes, 1 and 3 hours. The signal was detected as green vesicles distributed in the cytosol. The nucleus was stained with DAPi (blue). Figure 2a,b shows that in 1 mg/mL FITC-BSA-treated cells for 30 minutes, the fluorescence intensity was significantly lower at 4 °C in comparison to 37 °C cells [(AU) 4 °C: 1.67 ± 0.10 (n = 9) *versus* 37 °C: 3.62 ± 0.27(n = 20), **p < 0.01], indicating that the albumin internalization is temperature-dependent. The intracellular FITC-BSA quantification indicated that the albumin internalization process at 37 °C followed a significantly time-dependent trend [(AU) 30 minutes: 3.62 ± 0.27 *versus* 1 hour: 5.27 ± 0.42 (n = 20), ^##^p < 0.01]. Furthermore, the fluorescent signal did not change between 1 and 3 hours [(AU) 4.37 ± 0.46 (n = 11)], and it was not evidenced after 24 hours of stimulation, despite the presence of the cellular layer.

### Intracellular albumin overload leads to ER stress and PKC-δ phosphorylation in podocytes

**Figure 3:**
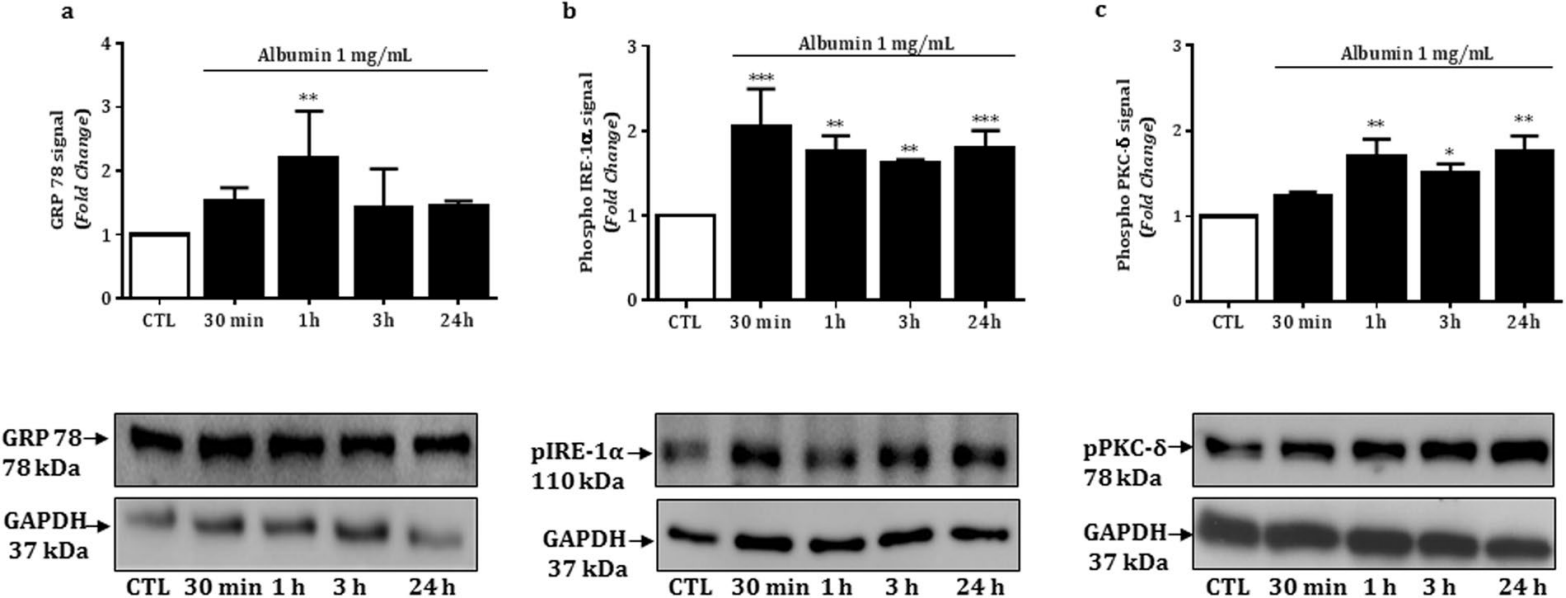
Relative expression and representative bands of GRP 78 (**a**) phosphorylated IRE1-α (**b**) and phosphorylated PKC-δ (**c**), in the control and treated podocytes. GAPDH was used as an internal control; the values (fold change from control) are mean ± SEM of 4 experiments.

We examined whether albumin overload triggers the ER stress response in podocytes. For these experiments, we used BSA without FITC. As shown in Fig. 3a,b and Table 2, the treatment with 1 mg/mL of BSA at 37 °C for 1 hour, but not for 30 minutes, 3 or 24 hours, induced significant increase in the expression of the chaperone GRP 78/BiP compared to the control group. The increased GRP 78 expression was parallel to the increase of phosphorylated IRE1-α expression, which was extended throughout the other experimental periods.

**Table 2:** Proteins expression in control and treated podocytes.

Proteins, fold change	Control	Alb −1 mg/ml			
		30 min	1 h	3 h	24 h
GRP 78	1.0 ± 0.0 (4)	1.54 ± 0.09 (4)	2.21 ± 0 0.41^**^(3)	1.41 ± 0.30 (4)	1.45 ± 0.03 (4)
Phospho IRE-1α	1.0 ± 0.0 (4)	2.06 ± 0.21 ^***^ (4)	1.76 ± 0.09^**^ (4)	1.63 ± 0.01^**^ (4)	1.80 ± 0.10^***^ (4)
Phospho PKC-δ	1.0 ± 0.0 (5)	1.23 ± 0.50 (5)	1.70 ± 0.20^**^ (4)	1.51 ± 0.09^*^ (5)	1.75 ± 0.18 ^**^ (4)
Phospho P38MAPK	1.0 ± 0.0 (4)	1.25 ± 0.10 (4)	2.15 ± 0.19^***^ (4)	3.47 ± 0.26^***^ (4)	2.78 ± 0.12 ^***^ (3)
Cleaved Caspase 12	1.0 ± 0.0 (4)	1.23 ± 0.06 (4)	1.46 ± 0.13^*^ (4)	2.43 ± 0.18^***^ (3)	1.62 ± 0.14 ^**^ (3)

The values are mean ± SEM; Number of experiments in parentheses. ^***^p < 0.001, ^**^p < 0.01 and ^*^p < 0.05 *versus* control (CTL), Alb, albumin; min, minutes; h, hours.

In addition to the ER stress marker, the treatment of podocytes with 1 mg/mL of BSA for 1, 3 or 24 hours, but not for 30 minutes, induced significant increase in the expression of phosphorylated PKC-δ compared to control group (Fig. 3c and Table 2).

### p38MAPK and caspase-12 mediate albumin-induced podocyte apoptosis

**Figure 4:**
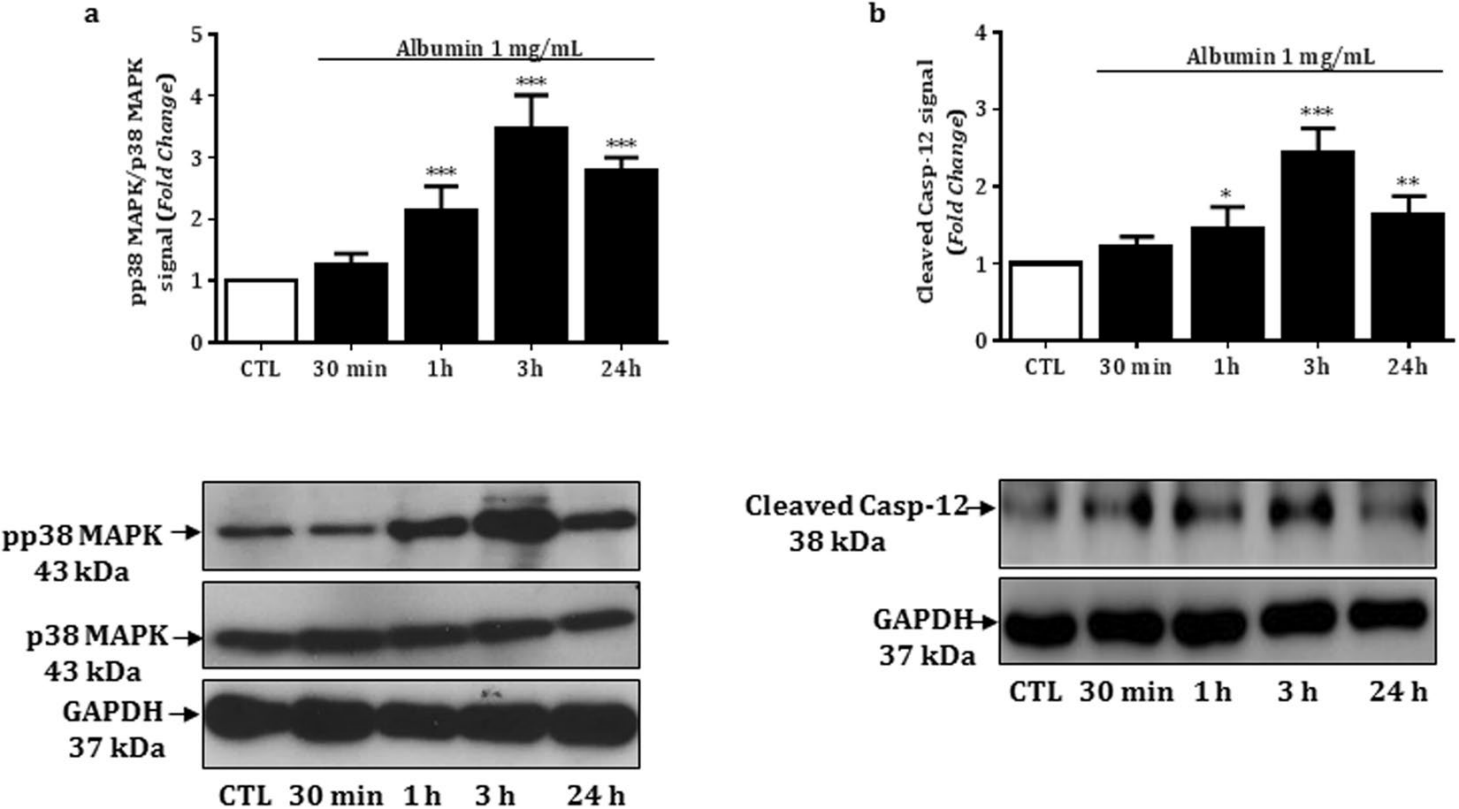
Relative expression and representative bands of non-phosphorylated and phosphorylated p38MAPK (**a**) or cleaved caspase 12 (**b**) in control and BSA (1 mg/mL)-treated podocytes for 30 minutes, 1, 3 or 24 hours. GAPDH was used as the internal control; the values (fold change from control) are represented as mean ± SEM of 3–4 experiments.

Considering that both IRE1-α and PKC-δ can mediate apoptosis through activation of the mitogen-activated protein kinase pathway^19,20^, we next evaluated the expression of the phosphorylated p38MAPK. As shown in Fig. 4a and Table 2, podocytes treated with BSA (1 mg/mL) at 37 °C for 1, 3 and 24 hours, but not for 30 minutes, showed a significant increase of phosphorylated p38MAPK protein expression compared to control group. In addition, the stimulatory effect of albumin on the expression of phosphorylated p38MAPK for all treatment periods is closely related with the increase of cleaved caspase 12 expression (Fig. 4b and Table 2).

**Figure 5:**
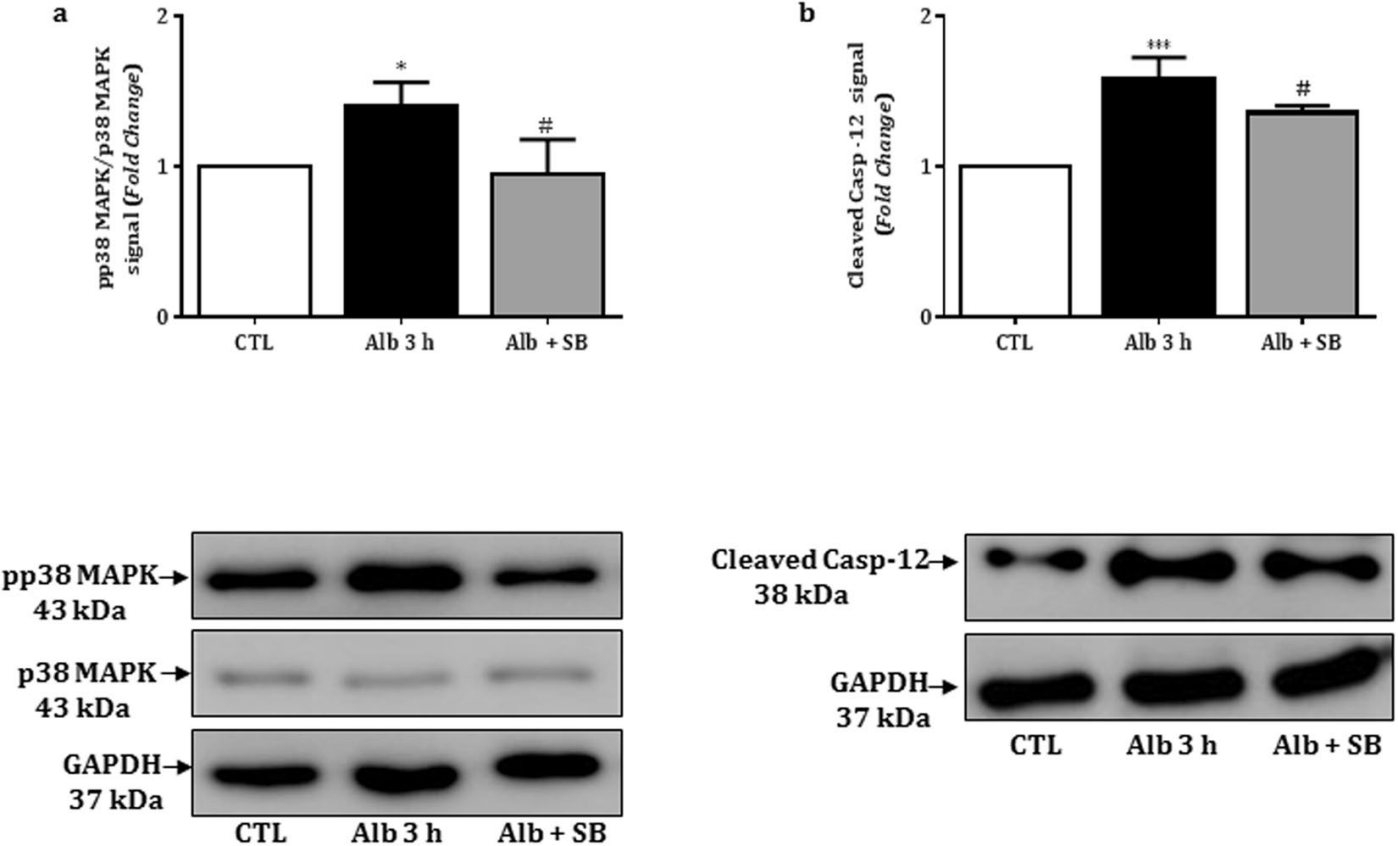
Relative expression and representative bands of non-phosphorylated and phosphorylated p38MAPK (**a**) or cleaved caspase 12 (**b**) in control and BSA (1 mg/mL) and/or SB203580-co-treated podocytes for 3 hours. GAPDH was used as the internal control; the values (fold change from control) are presented as mean ± SEM of 3–4 experiments.

Because the stimulatory effect of albumin on phosphorylated p38MAPK and cleaved caspase 12 expression reached the maximum in 3 hours, we next co-treated the cells with albumin 1 mg/mL and/or SB203580 (0.1 μM) at 37 °C for 3 hours. In this condition, SB203580 significantly reduced the stimulatory effect of albumin on both phosphorylated p38MAPK and cleaved caspase 12 expression (Fig. 5a,b and Table 3).

**Table 3:** Proteins expression in control and 1 mg/ml albumin and/or SB203580 (0.1 µM) treated podocytes for 3 hours.

Proteins, fold change	Control	Alb 3 h	Alb + SB 3 h
Phospho P38MAPK	1.0 ± 0.0 (4)	1.40 ± 0.09^*^ (3)	0.95 ± 0.11^#^ (4)
Cleaved Caspase 12	1.0 ± 0.0 (4)	1.58 ± 0.07^***^ (4)	1.36 ± 0.02^#^ (3)

The values are means ± SEM Number of experiments in parentheses. ^***^p < 0.001, ^*^p < 0.05 *versus* control (CTL); ^#^p < 0.05 *versus* Alb (albumin); min, minutes; h, hours.

## Discussion

In the present *in vitro* study, we demonstrated that albumin in a concentration- and time-dependent manner induces podocyte injury and apoptosis. These changes are associated with intracellular albumin overload, ER stress, upregulation of PKC-δ, p38MAPK and caspase 12.

Micropuncture studies in rats demonstrated that under physiological conditions, the albumin concentration in Bowman’s space is approximately 22.9 µg/mL^6,21^ and that this value can increase approximately 40-fold in nephropathy states^22^. On the other hand, *in vitro* studies revealed that in proximal tubular cells albumin endocytosis (range of 100 µg/mL to 1 mg/mL) was effective within 30 minutes^23^. However, the exposition of human podocytes to extracellular high concentrations of albumin (range of 5 to 10 mg/mL) for 24 and 48 hours led to increased cell death^24,25^. In the current study, using flow cytometry, we observed that prolonged exposure (24 hours) of immortalized mouse podocytes to albumin 1 mg/mL resulted in increased apoptosis. However, 5 and 10 mg/mL albumin failed to induce apoptosis. In part, our results differ from those obtained by Yoshida *et al*.^25^, possibly due to differences in our experimental design, including time of stimulation and evaluation method, in addition to the mechanism of saturation kinetics^26^.

The albumin internalization mechanisms in podocytes have been the main target of several studies, especially for the relative contribution of the multiple pathways involved in endocytosis process, which remain to be clarified. Endocytosis process can occur through non-specific binding of the molecule to the cell membrane and its subsequent internalization, or may be mediated by specific receptors^27^. Although the albumin endocytosis pathway has not been the focus of the current study, in a previous experimental trial, we observed that mouse podocyte express protein such as clathrin, caveolin-1, V-ATPase E-subunit, megalin and cubilin, which have been demonstrated as mediators of albumin endocytosis in kidney cells^28–31^. However, for the immunofluorescence analysis, we used an accurate method described by Bitsikas *et al*.^32^, which count overlap of different staining patterns, and we did not observe significant overlap of FITC-BSA and each protein investigated (data not shown), suggesting that in our experimental model the albumin internalization process can be mediated by other ways, including free fatty acids (FFAs) bound to albumin, G-protein-coupled receptors and Ras-related C3 botulinum toxin substrate 1 (RAC1)^33^.

Given the apoptotic effect of albumin, we focused our attention on the intracellular albumin overload-mediated podocyte apoptosis signaling in a time-dependent manner. Incubating podocytes with FITC-BSA (1 mg/mL), we observed that albumin-containing vesicles were evidenced in 30 minutes, 1 h and 3 h, but not after 24 hours of treatment. The time-dependent albumin overload occurred in parallel with apoptosis.

It is known that part of the albumin-containing vesicles in podocyte migrate through the cell body to lysosomal degradation^3^. However, persistent intracellular albumin overload may perturb cellular functions through reactive oxygen species generation and ER stress^34^. ER is the site of synthesis and folding of the secreted, membrane-bound and organelle-targeted proteins. Thus, the accumulation of unfolded and/or misfolded proteins in the ER can be referred to as ER stress^35^. In this condition, ER membrane protein kinase R (PKR)-like ER kinase (PERK), inositol-requiring enzyme 1 (IRE1) and activating transcription factor 6 (ATF6) activate the unfolded protein response (UPR). Under physiological conditions, the ER stress sensor proteins such as PERK, IRE1 and ATF6 interact with the chaperone GRP 78, which suppresses their activity. However, in response to ER stress, GRP 78 is recruited from ER stress sensor proteins to promote protein folding. In this condition, the ER stress sensor proteins are auto-activated and phosphorylate their target^36^. Using HEK293 cells, Zhu *et al*.^15^ reported that in response to ER stress, activated IRE1-α recruits the adaptor molecule TNF-receptor-associated factor 2 (TRAF2) to the ER membrane and activates the c-Jun N-terminal kinase (JNK). In addition, it is known that in *in vivo* and *in vitro* studies, the PKC-δ participates in ER-stress-induced apoptosis, primarily activating the JNK pathway^20,37–39^. In the current study, the treatment of podocytes with BSA 1 mg/mL for 1 hour resulted in increased GRP 78, phosphorylated IRE1-α and PKC-δ expression. Together, these results indicate that the GRP 78/IRE1-α/PKC-δ signaling pathway contributed to ER stress and induction of albumin-overload-dependent podocyte injury. Additionally, we observed that with 1 mg/mL BSA, the increase of phosphorylated IRE1-α expression (30 min) was prior to the increase of phosphorylated PKC-δ expression, which was significantly increased in 1 hour, and both proteins sustained the phosphorylated states during the apoptotic process, confirming their pivotal role in the cell death. Given that the phosphorylation of PKC-δ is associated with ER stress response in podocytes treated with albumin, it is important to understand the key mechanisms by which PKC-δ initiates and maintains the apoptotic process.

**Figure 6:**
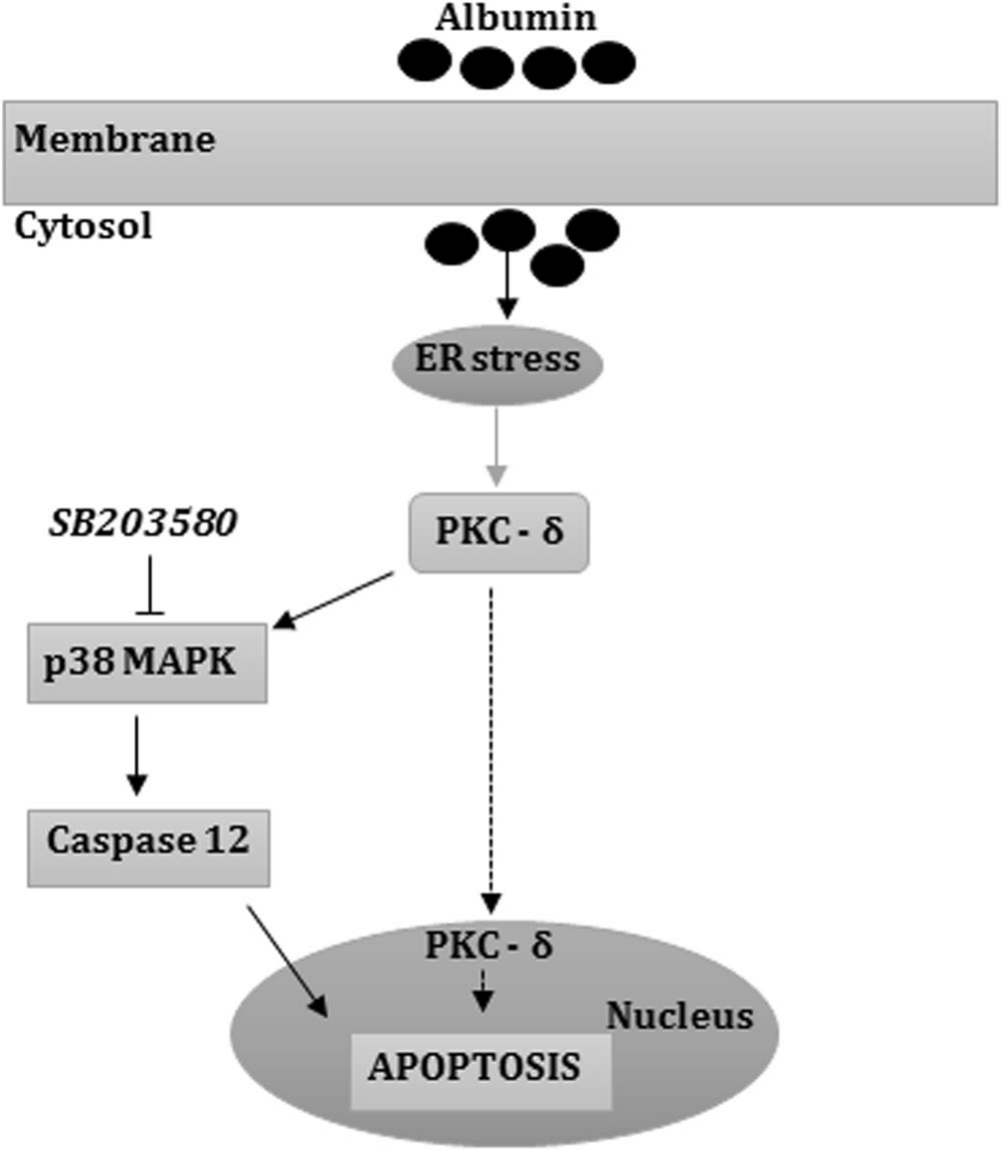
Proposed model for albumin-induced podocyte apoptosis. The intracellular albumin overload leads to ER stress, activating the GRP 78/IRE1-α pathway and the subsequent phosphorylation of PKC-δ. Both IRE1-α and PKC-δ may phosphorylate the SB203580-sensitive p38MAPK, which cleaves caspase 12, resulting in podocyte apoptosis.

We have recently demonstrated that both PKC-δ and p38MAPK are involved in the angiotensin-II-mediated podocyte apoptosis process^40^. In addition, PKC-δ has been shown to interact with several members of the mitogen-activated protein kinase (MAPK) family, including p38MAPK^20^ and NH2 terminal kinase (JNK)^20,39^. Other *in vitro* studies reported that podocytes from patients with nephrotic-range proteinuria or podocytes from mice exposed to albumin, resulted in p38MAPK-mediated apoptosis^25,41^. However, the pathway linking p38MAPK and podocyte injury was not demonstrated. Our data revealed that albumin-mediated expression of phosphorylated p38MAPK and cleaved caspase 12, as well as podocyte apoptosis, was prevented by SB203580. Together, these results reinforce the role of the p38MAPK/caspase 12 interaction on albumin-induced podocyte apoptosis. Thus, our data provide new evidences that albumin-mediated podocyte apoptosis involves complex intracellular pathways including GRP 78/IRE1-α/PKC-δ/p38MAPK/caspase 12 activation (Fig. 6).

## Conclusion

ER stress conditions have been observed in numerous pathological events including cardiovascular and renal diseases. Despite its importance in pathological conditions, several complex questions remain regarding ER-stress-induced apoptosis. Given that ER stress mediators are well defined and specific, they could be useful targets for therapy. Therefore, our current study demonstrated a crosstalk between albumin overload/ER stress/podocyte apoptosis and identified new targets for the prevention of podocyte injury in nephropathies associated with albuminuria.

## Materials and Methods

### Cell culture

The immortalized mouse podocytes were developed by Prof. Dr. Karlhans Endlich, University of Heidelberg, Germany and kindly provided by Prof. Dr. Niels Olsen Saraiva Camara, Institute of Biomedical Sciences, University of Sao Paulo. As previously described^42^, the cell culture was grown in 75-cm^2^ flasks (Corning, New York, NY, USA) coated with type I collagen and maintained in RPMI-1640 medium (Thermo Fisher Scientific INC, St Peters, MO, USA) supplemented with 10% fetal bovine serum (FBS, Thermo Fisher Scientific), 30 IU/mL IFN-γ (Cell Sciences, Newburyport, MA, USA), 100 IU/mL penicillin, 100 μg/mL streptomycin and 2 mmol/L L-glutamine (pH 7.4 ) at 33 °C in a 5% CO_2_ atmosphere. The culture medium was changed every two days until 85% confluence. Cells at passages 8 to 10 were detached by incubation with 5 mL trypsin-EDTA solution (Thermo Fisher Scientific, 0.05%) for 5 min at 37 °C. The cells were seeded for differentiation at specific cell densities into cell culture dishes with a diameter of 100 mm × 20 mm (Corning) in the same conditions, except for the absence of IFN-γ. The cells were differentiated for 10–15 days. Podocyte differentiation was confirmed by the identification of podocin and synaptopodin on protein expression. Phalloidin staining was used to identify the podocyte cytoskeleton.

### Experimental design

Differentiated podocytes were distributed in control and experimental groups. In control or treated cells, albumin internalization was examined by immunofluorescence images using FITC-BSA (Sigma Aldrich), and the apoptosis levels were assessed by flow cytometry. The protein expression was evaluated by immunoblotting. For p38MAPK inhibition, the cells were previously treated with SB203580, a specific p38MAPK inhibitor (0.1 μM, Merck Millipore, Temecula, CA, USA), for 1 hour, followed by BSA (1 mg/mL) plus SB203580 for additional 3 hours. For the culture medium and experimental solutions, the osmolality was 290 mOsm/kg H_2_O.

### Annexin V/7-AAD staining and flow cytometry

As previously described^42^, control and treated podocytes were trypsinized, and 3 mL of BioLegend Cell Staining Buffer (BioLegend, San Diego, CA, USA) was added to the cell samples. The cell suspensions were centrifuged at 2500 rpm for 5 minutes. This procedure was repeated once more, followed by suspension of the cells in Annexin V Binding Buffer (200 µL, BioLegend). 1 × 10^5^ cells/mL were transferred to a cytometry sample tube and 0.4 μL of FITC-Annexin V was added. Then, 0.6 μL of 7-Aminoactinomycin −7-AAD (1 mg/mL, Thermo Fisher Scientific) was added. The samples were incubated in the dark for 10 min. Cell fluorescence was subsequently analyzed using a BD FACSCanto II Flow Cytometer (San Jose, CA, USA), calibrated to detect 10.000 events. Cells positive for FITC -Annexin V and 7-AAD were considered apoptotic. The values are shown as percentage (%).

### Albumin internalization

To evaluate albumin internalization, podocytes cultured on glass coverslips were treated with FITC-BSA (1 mg/mL in serum free RPMI-1640 medium) at 4 °C or 37 °C for 30 minutes; 37 °C for 1 and 3 hours. Cells were then fixed with 4% paraformaldehyde in PBS (0.15 M NaCl containing 10 mM sodium phosphate buffer, pH 7.4) for 4 minutes and permeabilized with 0.1% Triton X-100 in PBS for an additional 5 minutes. Next, the glass coverslips were washed three times with PBS at room temperature, stained with 4′6-diamidine-2′-phenylindole dihydrochloride (DAPI; Sigma Aldrich) for 5 minutes and the fluorescence signal was examined with a Zeiss LSM 788 confocal microscope equipped with 40x and 63x objective Plan-Apochromat, zoom factor 1, using a laser excitation of 488 nm to FITC-BSA acquisition and 405 nm to DAPI acquisition. The fluorescence signal is shown as fluorescence intensity (arbitrary units) and quantified using the ImageJ software (National Institute of Mental Health, Bethesda, MD).

### Immunoblotting

Total proteins from control or treated podocytes were extracted using ice-cold RIPA buffer (Merck Millipore) with protease and phosphatase inhibitors (Sigma Aldrich). Immunoblot analysis was performed on aliquots containing 30 μg/lane of proteins resolved in 8–10% SDS-PAGE as previously described^43,44^. The separated protein samples were transferred to a polyvinylidene fluoride (PVDF) membrane. After blocking with 5% albumin for 1 hour, the blots were probed in the same buffer overnight at 4 °C with specific primary antibodies. Next, the blots were washed four times with 1% Tris-buffered saline (TBS; 50 mM Tris-HCl, 150 mM NaCl, pH 7.5) plus 0.05% Tween-20 (TBST), and incubated with recommended dilutions of secondary antibodies for 1 hour, followed by four washes and treatment with Enhanced Chemiluminescence (ECL) reagent (GE HealthCare, Aurora, OH, USA). The bands were quantified by optical densitometry using the ImageJ software (National Institute of Mental Health). Protein expression was quantified as the ratio of a specific band to glyceraldehyde-3-phosphate dehydrogenase (GAPDH, Cell Signaling, #2118). The following primary antibodies were used in this study: rabbit anti-podocin (1:3000, Sigma Aldrich, #P0372); rabbit anti-synaptopodin (1:4000, Santa Cruz Biotechnology, #SC50459 Dallas, TX, USA); rabbit anti-chaperone GRP 78 (1:8000, #SPC167 StressMarq, Victoria, BC, CA); rabbit anti-phospho IRE1-α (1:3000, #AB48187 Abcam, Cambridge, MA, USA); rabbit anti-phospho PKC-δ (1:3000, #AB5658-50, Abcam); rabbit anti-p38MAPK and anti-phospho-p38MAPK (1:2000, #9212 and #4511, Cell Signaling); rabbit anti-caspase 12 (1:4500, #AB62484, Abcam, Cambridge, MA, USA) and rabbit anti-GAPDH (1:2000, #2118Cell Signaling). A horseradish peroxidase-conjugated goat secondary antibody against rabbit (#111-035-144, Jackson ImmunoResearch Laboratories, Baltimore, MD, USA) was additionally used. The values are represented as fold change compared to the control group.

### Statistical analysis

The data are reported as the mean ± SEM. For comparisons among the groups, a one-way ANOVA followed by Bonferroni’s test (GraphPad Prism 6 Software, San Diego, USA) was performed. The differences with p < 0.05 were considered statistically significant.

### Ethics approval

All of the experimental protocols were conducted in accordance with the ethical standards approved by the Institutional Animal Care and Use Committee of the University of Sao Paulo (Protocols no. 792/2015).

## Supplementary information

Supplementary dataset 1

## Data Availability

All data generated or analyzed during this study are included in this published article (and its Supplementary Information Files).
