# Supplementary material for: Intracellular albumin overload elicits endoplasmic reticulum stress and PKC-delta/p38 MAPK pathway activation to induce podocyte apoptosis
Source: Sci Rep. 2018 Dec 20;8:18012. doi: 10.1038/s41598-018-36933-9 (PMC6301950; doi:10.1038/s41598-018-36933-9)
Supplement: Supplementary file 1 — Supplementary dataset 1 [file 41598_2018_36933_MOESM1_ESM.pdf]

Intracellular albumin overload elicits endoplasmic reticulum stress and upregulation of the PKC-delta/p38 MAPK pathway to induce podocyte apoptosis

Manuscript number SREP-18-12502A

[1]Guilherme Lopes Gonçalves, [1]Juliana Martins Costa-Pessoa, [1]Karina Thieme, [1]Bruna Bezerra Lins, and [1]Maria Oliveira-Souza

Raw Data Figures

Figure 1 a : Podocin and Synaptopodin protein expression

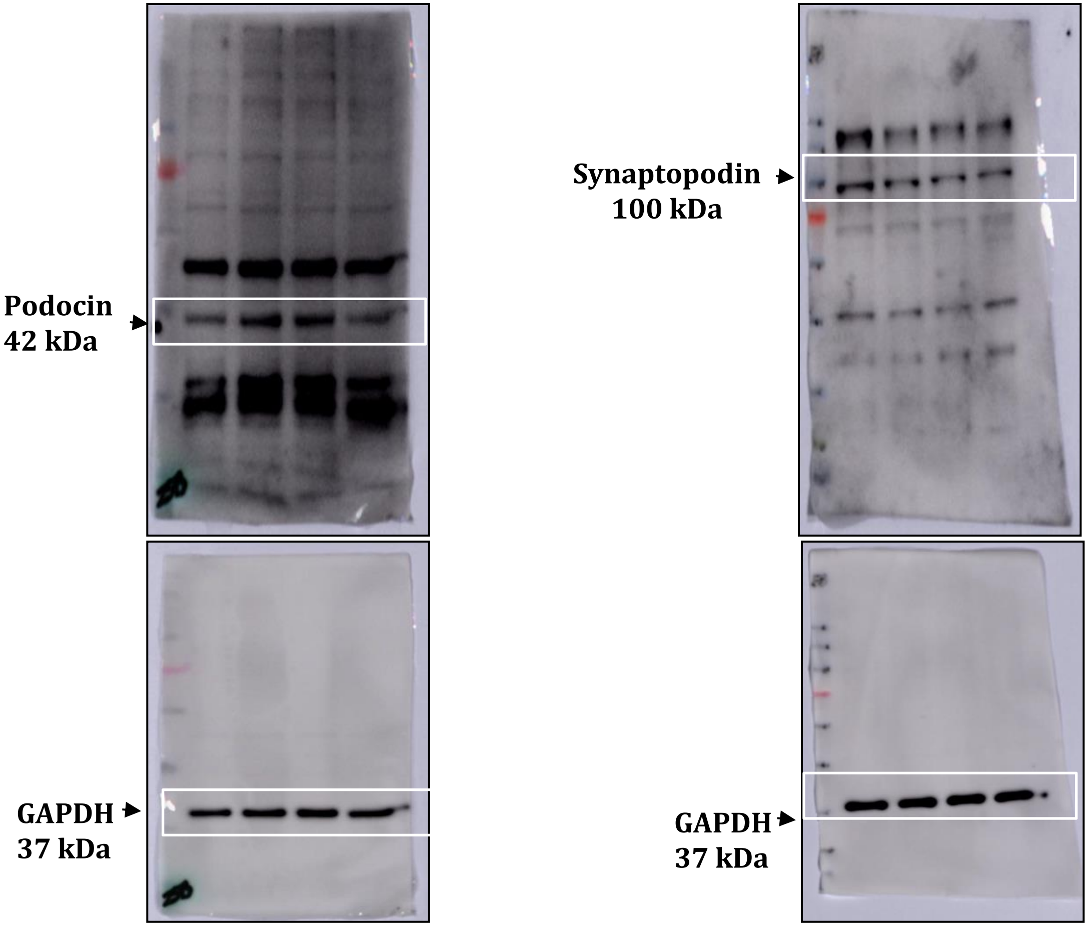

Suplementary Figure 1 a: Raw data of podocin and synaptopodin protein expression, as well as the internal control glyceraldehyde-3-phosphate dehydrogenase (GAPDH).

Figure 2 a: FITC-albumin did not internalize at 4ºC

4ºC for 30 minutes

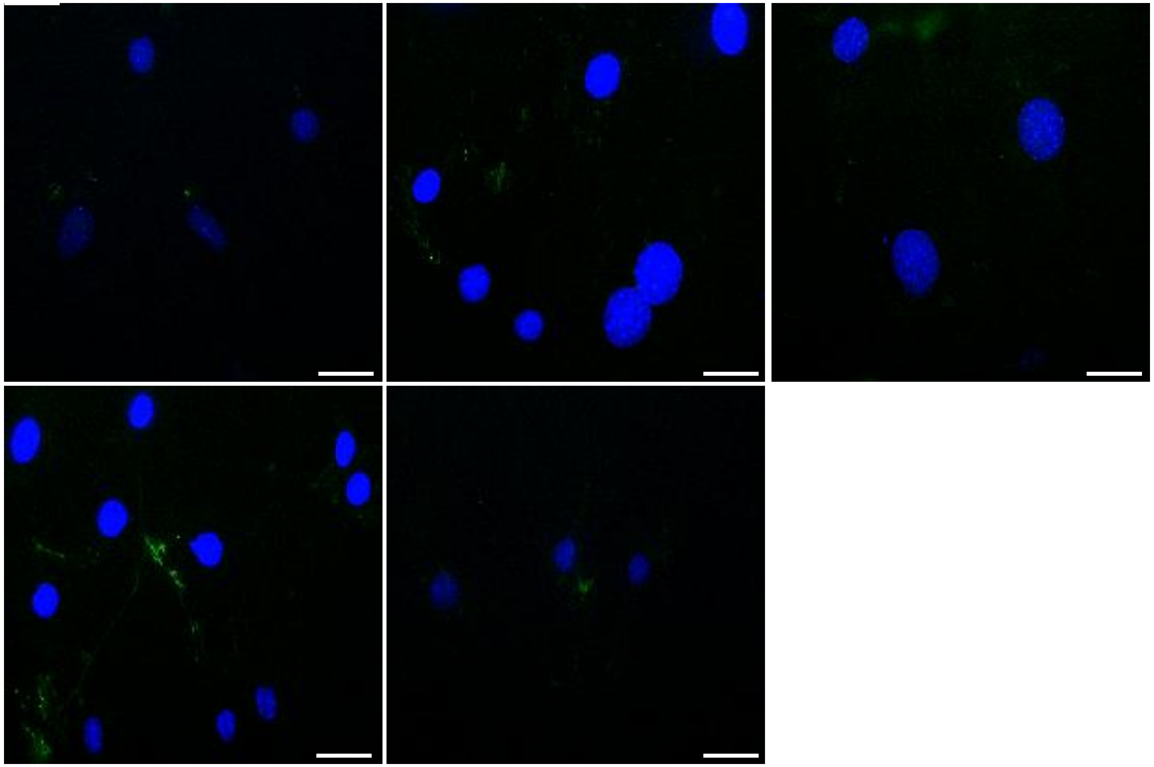

Suplementary Figure 2 a: Raw data of fluorescent images demonstrating that at 4ºC for 30 minutes , albumin did not internalizes in podocytes .The signal was detected only as blue nucleus (DAPI), 40x objetive. Bar, 20 µm.

Figure 2 a: FITC-albumin internalizes at 37ºC for 30 minutos

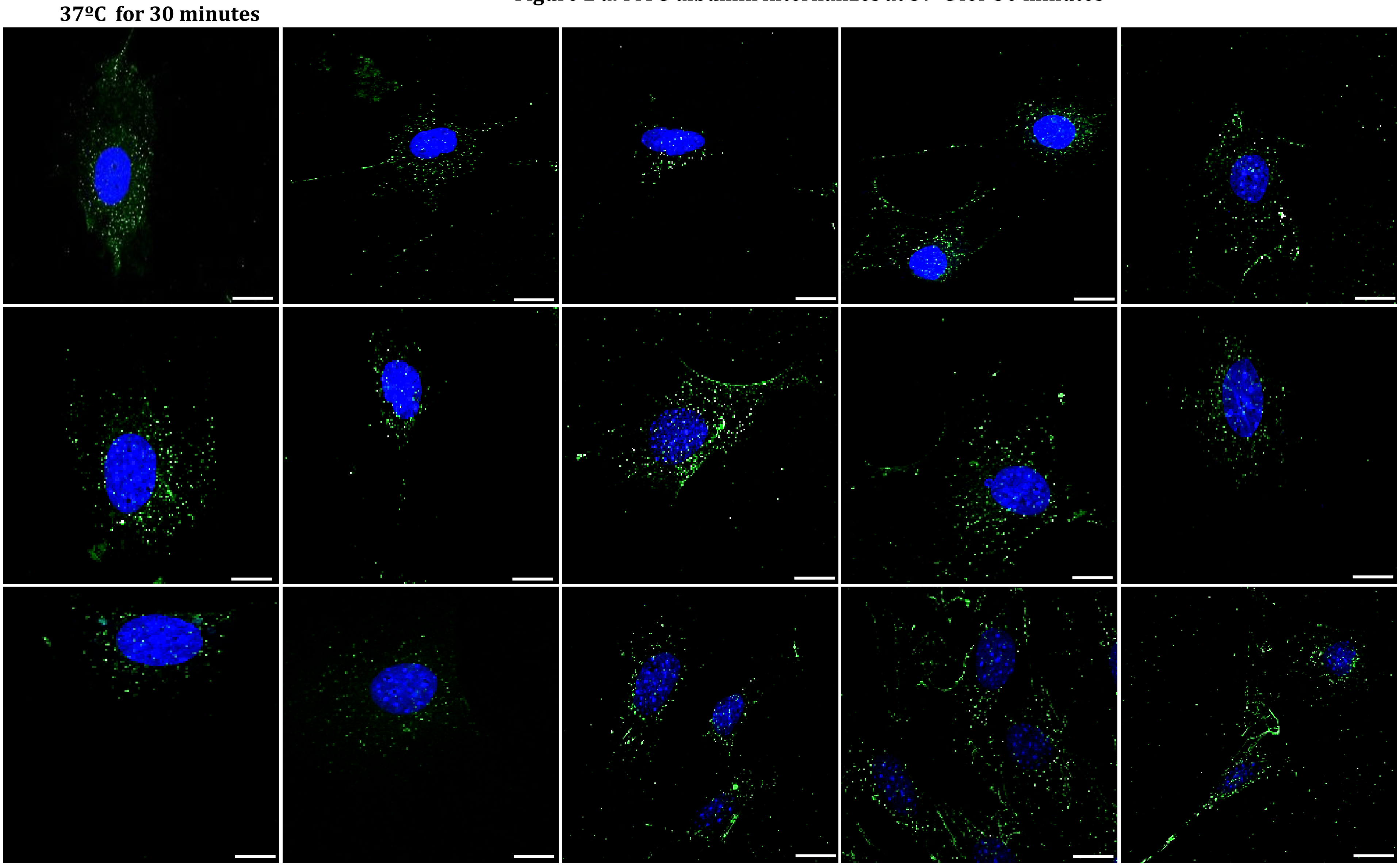

Suplementary Figure 2 a: Raw data of fluorescent images demonstrating that at 37ºC for 30 minutes, albumin internalizes in podocytes. The signal was detected as green vesicles distributed in the cytosol and blue nucleus (DAPI), 63x objetive. Bar, 20 µm.

Figure 2 a: FITC-albumin internalizes at 37ºC for 1 hour

37ºC for 1 hour

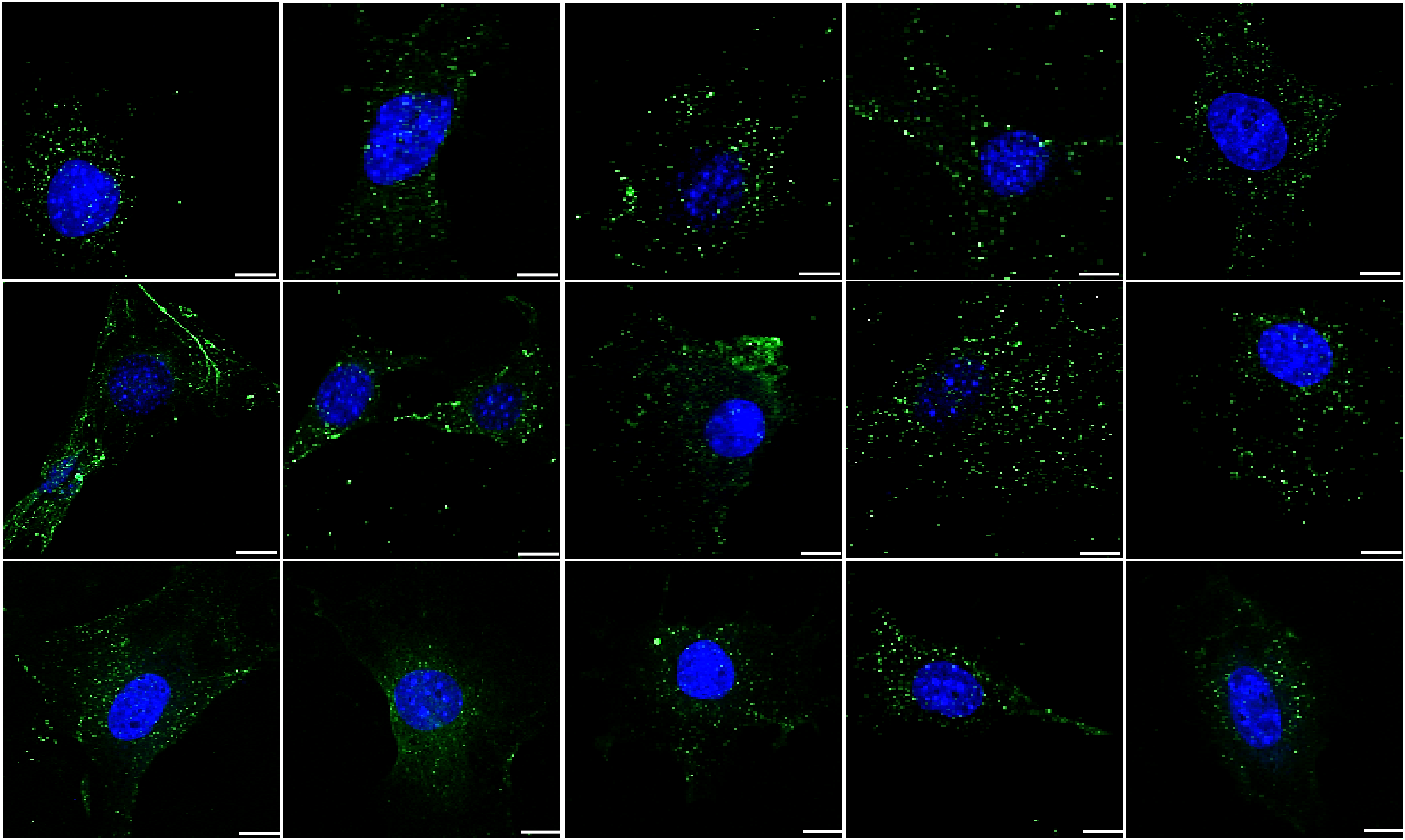

Suplementary Figure 2 a: Raw data of fluorescent images demonstrating that at 37ºC for 1 hour, albumin internalizes in podocytes. The signal was detected as green vesicles distributed in the cytosol and blue nucleus (DAPI), 63x objetive. Bar, 20 μm.

Figure 2 a: FITC-albumin internalizes at 37°C for 3 hours

37ºC for 3 hours

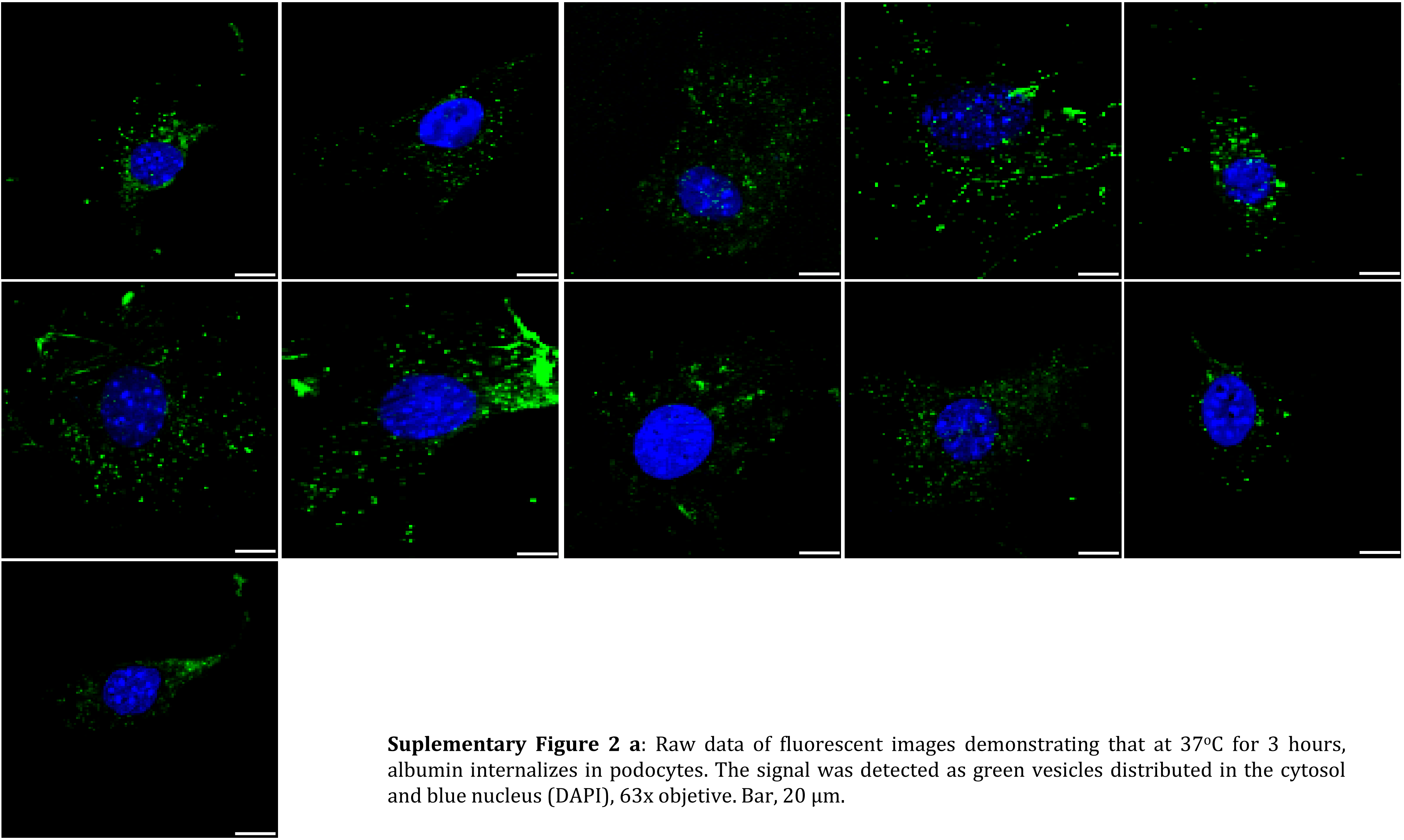

Suplementary Figure 2 a: Raw data of fluorescent images demonstrating that at 37°C for 3 hours, albumin internalizes in podocytes. The signal was detected as green vesicles distributed in the cytosol and blue nucleus (DAPI), 63x objetive. Bar, 20 μm.

Figure 3: GRP 78 phosphorylated IRE1-α and phosphorylated PKC-δ protein expression

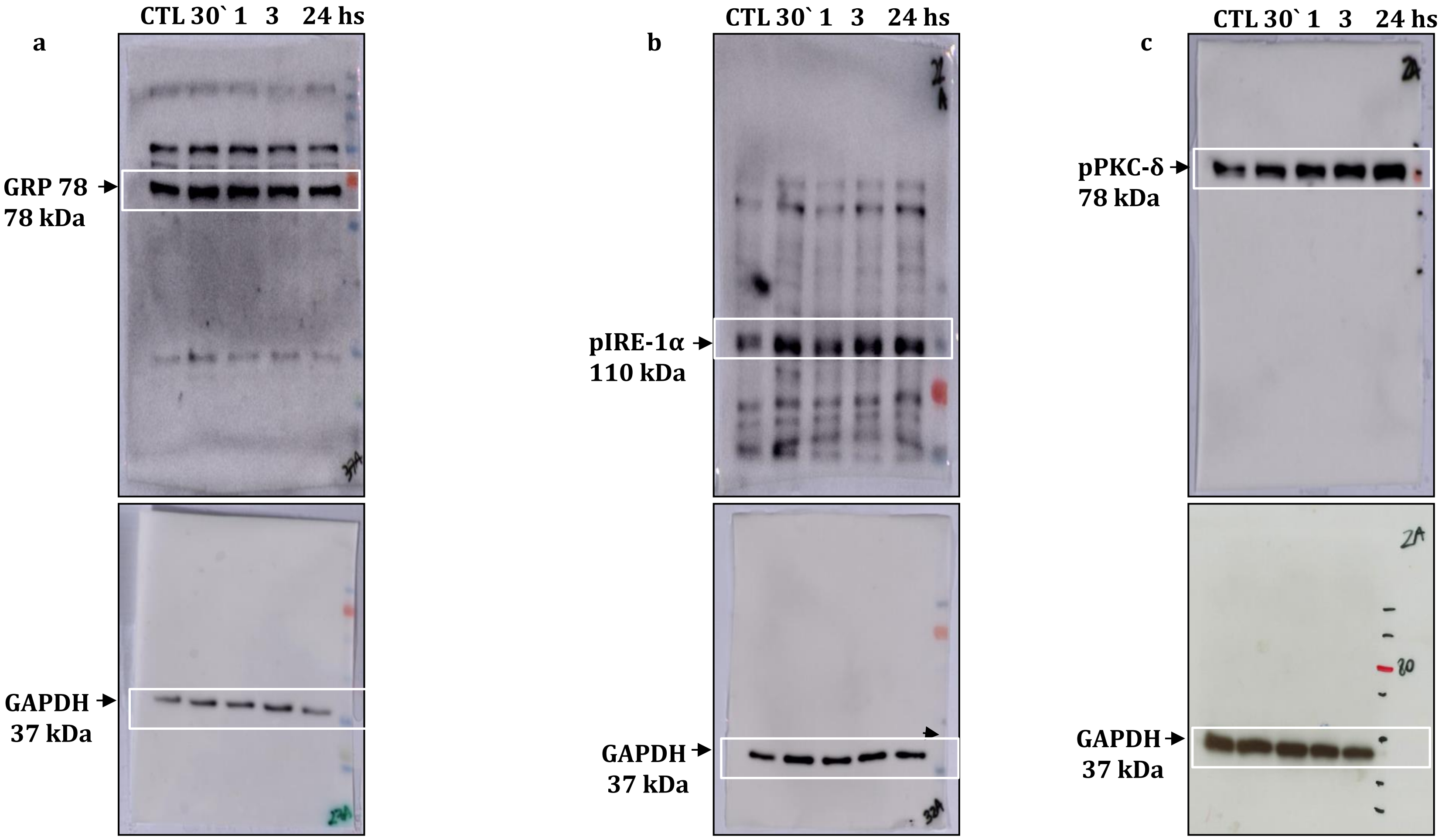

Suplementary Figure 3: Raw data of GRP 78 (A) phosphorylated IRE1-α (B) and phosphorylated PKC-δ (C) protein expression, in the control and treated podocytes (37°C) for 30 minutes, 1, 3 and 24 hours. GAPDH was used as an internal control .

Figure 4: phosphorylated p38MAPK or cleaved caspase 12 protein expression

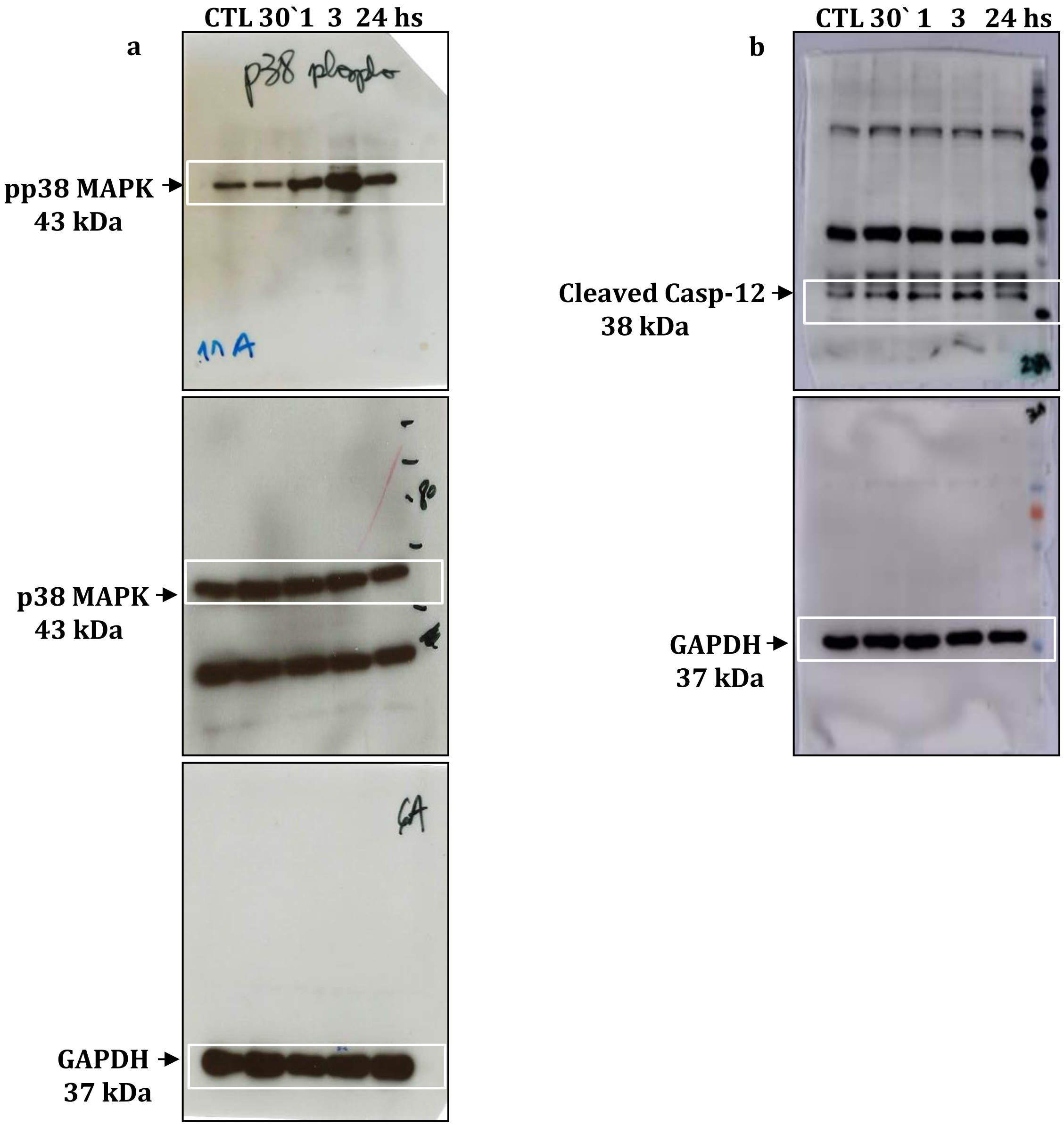

Suplementary Figure 4: Raw data of non-phosphorylated and phosphorylated p38MAPK (A) or cleaved caspase 12 (B) protein expression in the control and albumin (1 mg/mL)-treated podocytes (37°C) for 30 minutes, 1, 3 or 24 hours. GAPDH was used as the internal control.

Figure 5: p38MAPK and apoptosis

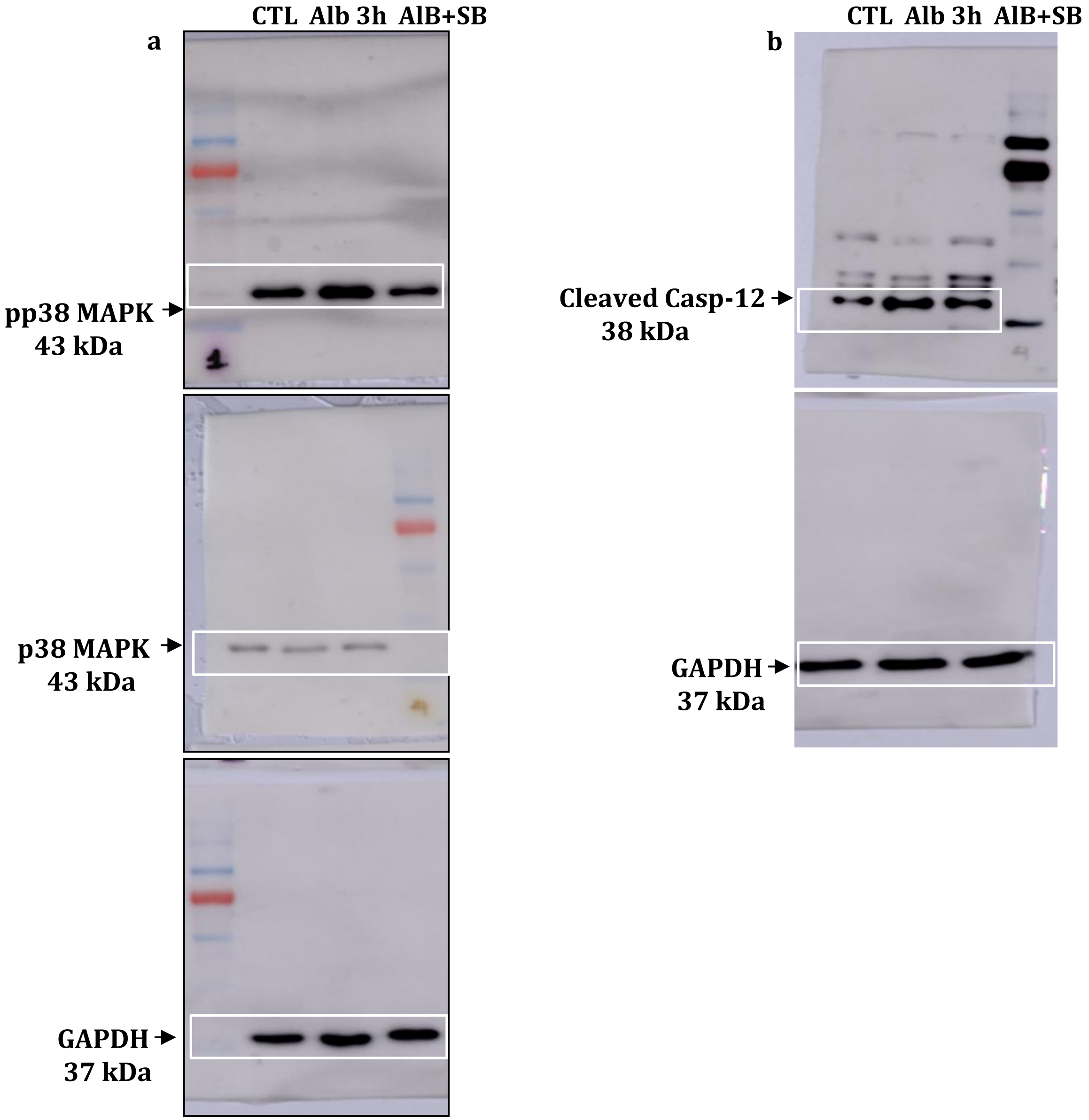

Suplementary Figure 5: Raw data of non-phosphorylated and phosphorylated p38MAPK (A) or cleaved caspase 12 (B) protein expression in the control and albumin (1 mg/mL) for 3 hours and/or SB203580 (0,1µM)-co-treated podocytes for 3 hours. GAPDH was used as the internal control.
